# Transcriptomic profiling of the flower scent biosynthesis pathway of *Cymbidium faberi* Rolfe and functional characterization of its jasmonic acid carboxyl methyltransferase gene

**DOI:** 10.1186/s12864-019-5501-z

**Published:** 2019-02-11

**Authors:** Qi Xu, Songtai Wang, Huazhu Hong, Yin Zhou

**Affiliations:** 1 Center of Applied Biotechnology, Wuhan University of Bioengineering, Wuhan, 430415 People’s Republic of China; 2 College of Bioscience and Biotechnology, Wuhan University of Bioengineering, Wuhan, 430415 People’s Republic of China; 3 Present Address: Hainan Key Laboratory for the Sustainable Utilization of Tropical Bioresources, College of Agriculture, Hainan University, Haikou, 570228 People’s Republic of China

**Keywords:** Transcriptome sequencing, Flower fragrance, Jasmonic acid carboxyl methyltransferase (JMT), Oriental orchids

## Abstract

**Background:**

*Cymbidium faberi*, one of the most famous oriental orchids, has a distinct flower scent, which increases its economic value. However, the molecular mechanism of the flower scent biosynthesis was unclear prior to this study. Methyl jasmonate (MeJA) is one of the main volatile organic compounds (VOC) produced by the flowers of *C. faberi*. In this study, unigene 79,363 from comparative transcriptome analysis was selected for further investigation.

**Results:**

A transcriptome comparison between blooming and withered flowers of *C. faberi* yielded a total of 9409 differentially expressed genes (DEGs), 558 of which were assigned to 258 pathways. The top ten pathways included α-linolenic acid metabolism, pyruvate metabolism and fatty acid degradation, which contributed to the conversion of α-linolenic acid to MeJA. One of the DEGs, jasmonic acid carboxyl methyltransferase (*CfJMT*, Unigene 79,363) was highly expressed in the blooming flower of *C. faberi*, but was barely detected in leaves and roots. Although the ectopic expression of *CfJMT* in tomato could not increase the MeJA content, the expression levels of endogenous MeJA biosynthesis genes were influenced, especially in the wound treatment, indicating that CfJMT may participate in the response to abiotic stresses.

**Conclusion:**

This study provides a basis for elucidating the molecular mechanism of flower scent biosynthesis in *C. faberi*, which is beneficial for the genetically informed breeding of new cultivars of the economically valuable oriental orchids.

**Electronic supplementary material:**

The online version of this article (10.1186/s12864-019-5501-z) contains supplementary material, which is available to authorized users.

## Background

Orchids (Orchidaceae family) are one of the largest families of monocotyledonous plants, and many members have great economic and ornamental value. After the publication of the whole-genome sequence of *Phalaenopsis equestris,* extensive studies have focused on the molecular mechanism of orchid development and interaction with microbes [1]. In contrast to the more broadly cultivated tropical species, the subtropical oriental orchids often have strong flower fragrance but do not have showy colors. Thus, the fragrance is the primary attribute of flower quality in these species.

Flower fragrance is determined by complex mixtures of volatile compounds, generally emitted from petals, sepals, and the gynostemium of orchids [2]. In plants, flower fragrance is mostly an adaptation for attracting insects for pollination or a defense against predation by herbivores [3]. There are more than 100 compounds of the flower fragrance in blooming flowers of *Cymbidium faberi*, one of the longest cultivated species of oriental orchids. Among these, methyl jasmonate (MeJA) is the most abundant, but is almost undetectable in the volatile fragrance of withered flowers [4, 5].

Jasmonic acid (JA), which is ubiquitously distributed in the plant kingdom, is synthesized via the octadecanoid pathway [6]. It is involved in diverse biological processes, including seed germination, flower and fruit development, leaf abscission, senescence and defensive responses against abiotic and biotic stresses [7, 8]. The biosynthetic pathway of JA in plants was elucidated previously, and its derivative MeJA was found to be formed by the action of jasmonic acid carboxyl methyltransferase (JMT), which transfers a methyl group derived from S-adenosyl-L- methionine to the carboxyl group of JA [9].

Studies on the function of JMT have been conducted in many plants, such as *Arabidopsis thaliana* (Cruciferae) [7], *Capsicum annuum* (Solanaceae) [10], *Glycine max* (Fabaceae) [11], *Populus trichocarpa* (Salicaceae) [12] and *Fragaria × ananassa* (Rosaceae) [13]. Most of the recent studies have provided insights into the function of JMT in the stress response, as well as the development of seeds, leaves, roots, flowers and fruits, and only rare studies investigated its role in the synthesis of flower fragrance, although MeJA was initially identified in the flower fragrance of *Jasminum grandiflorum*, as evidenced by its name [14].

Most recent studies on *Cymbidium* spp. were focused on the propagation of sterile seedlings [15–17], leaf and flower morphogenesis [18–20], and the characterization of the chemical components of flower scent [21–23]. Flower scent is a valuable economic trait for *Cymbidium* cultivars, but the complex composition of its components has retarded the studies on the molecular mechanisms of their biosynthesis.

With the rapid development of “omics” approaches, high-throughput studies have been applied to obtain enormous amounts of data for elucidating the metabolic pathways of different organisms as well as their underlying molecular mechanisms. Because RNA-seq enables the analysis of the transcripts of large numbers of active genes and complex metabolite networks in species without a clear genomic background, it has become a preferred method to guide the direction for extensive performance improvement [20, 24–27]. Since the main component of flower fragrance in *Cymbidium faberi* is MeJA, the blooming flowers and withered flowers were compared to screen out differentially expressed genes (DEGs) and related metabolic pathways by comparative transcriptome sequencing, focusing especially on flower scent biosynthesis. Furthermore, the full length of an up regulated gene in blooming flowers, *CfJMT*, was cloned and its spatio and temporal expression pattern was determined, revealing that it is expressed mainly in the blossom stage. The promoter sequence of *CfJMT* gene revealed many conserved cis-acting elements, including G-box motifs at-547, − 1088 and − 1493 bp, which is consistent with the expected regulation sites of several transcription factors. The *CfJMT* gene was also ectopically overexpressed in tomato. Although this overexpression did not measurably increase the content of MeJA, the responses to abiotic stresses of the transformants were changed. Following wounding, the transcription levels of MeJA-related genes in tomato were all increased several folds, suggesting that the *CfJMT* gene may participate in the resistance to abiotic stress in tomato.

## Results

### Evaluation of the RNA-seq datasets

The flower development of *C. faberi* can be divided into three stages: flower bud stage, blooming stage and withered stage. According to GC/MS analysis, one of the main VOCs of the flower fragrance of *C. faberi* in blooming stage is methyl jasmonate (MeJA), followed by alkanes and other esters, which were not detected in the withered stage, as shown in Additional file 1: Figure S1. Fresh flowers from the blooming and withered samples were selected for RNA extraction. After isolation, purification and detection, the RNA samples had an A260/280 of ~ 2.0, RIN ≥ 7, 28S/18S ≥ 1.5, and a concentration of ~ 1000 ng/μL, which fulfilled the requirements for RNA-seq.

The sequencing of two pools using an Illumina HiSeq 2500 platform yielded an average of 56.4 and 53.9 million reads in the three blooming (S1) and three withered flower samples (S2) of *C. faberi*, respectively. After eliminating adaptor sequences, approximately 55.7 and 53.5 million clean reads with 46% GC content were obtained, and at least 80% of the total sequences were of higher quality than Q30. The reads were then de novo assembled and yielded a total of 86,784 contigs with 773 bp average length and N50 of 1393 bp, of which 26,973 (31.1%) were annotated based on the five public databases, including Nr, UniProt, COG, GO and KEGG. Blast results showed that 30.2% of unigenes had extremely high homology to known sequences in the Nr database (E-value<10^− 100^), while 20.5% had high (E-value<10^− 50^) and 49.3% moderate homology (E-value<10^− 5^) (Additional file 1: Figure S2). The Nr species distribution showed that the top hits among the best matches were from *Phoenix dactylifera* (41%), followed by *Musa acuminata ssp. malaccensis* (14.6%), *Vitis vinifera* (4.3%), *Nelumbo nucifera* (3.7%), *Oryza sativa ssp. japonica* (1.8%) and *Theobroma cacao* (1.3%) (Additional file 1: Figure S3).

### Screening and annotation of DEGs and pathway analysis of unigenes, focusing on those associated with flower scent in *C. faberi*

**Fig. 1:**
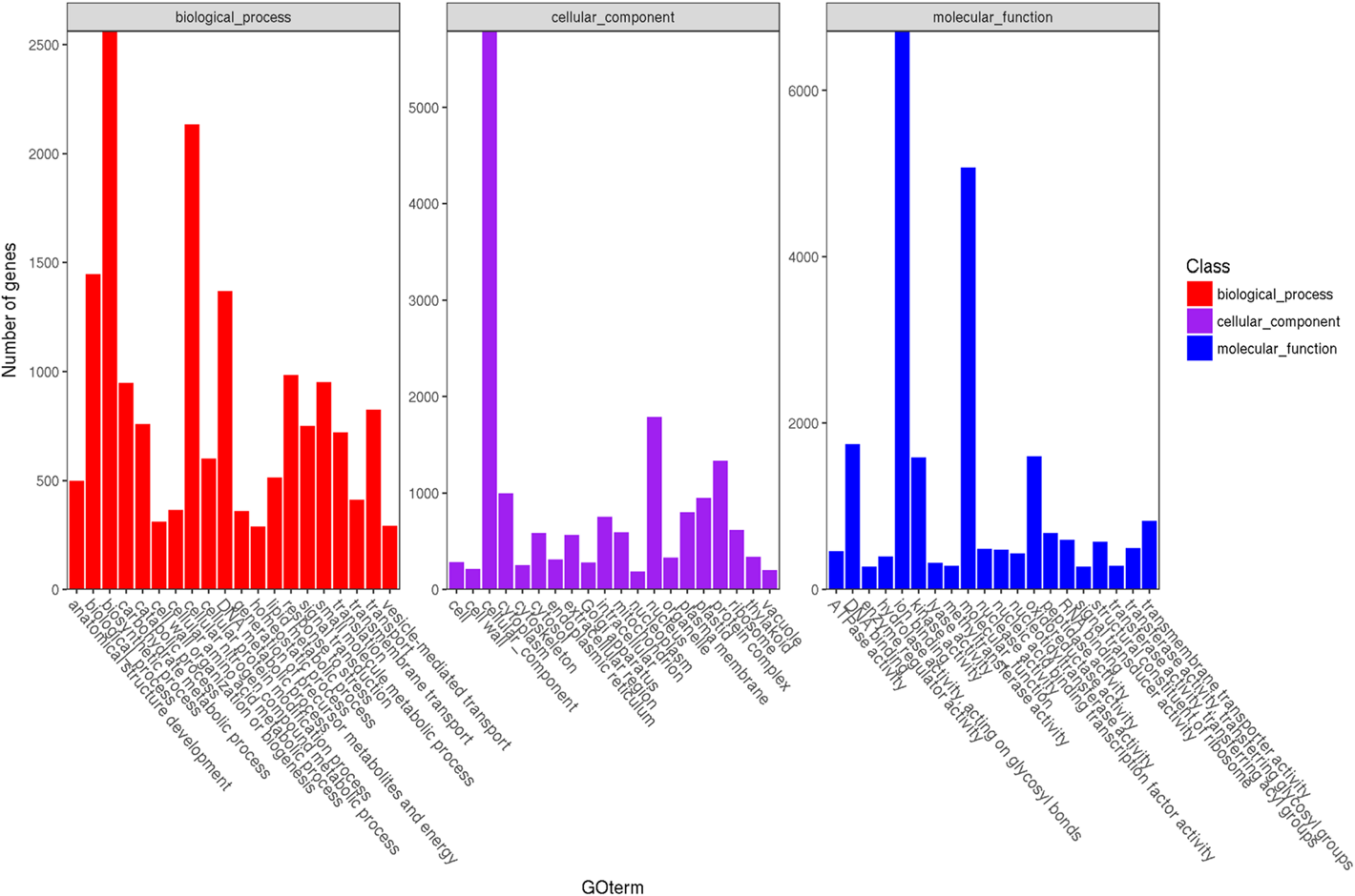
GO classification of *Cymbidium faberi* unigenes

**Fig. 2:**
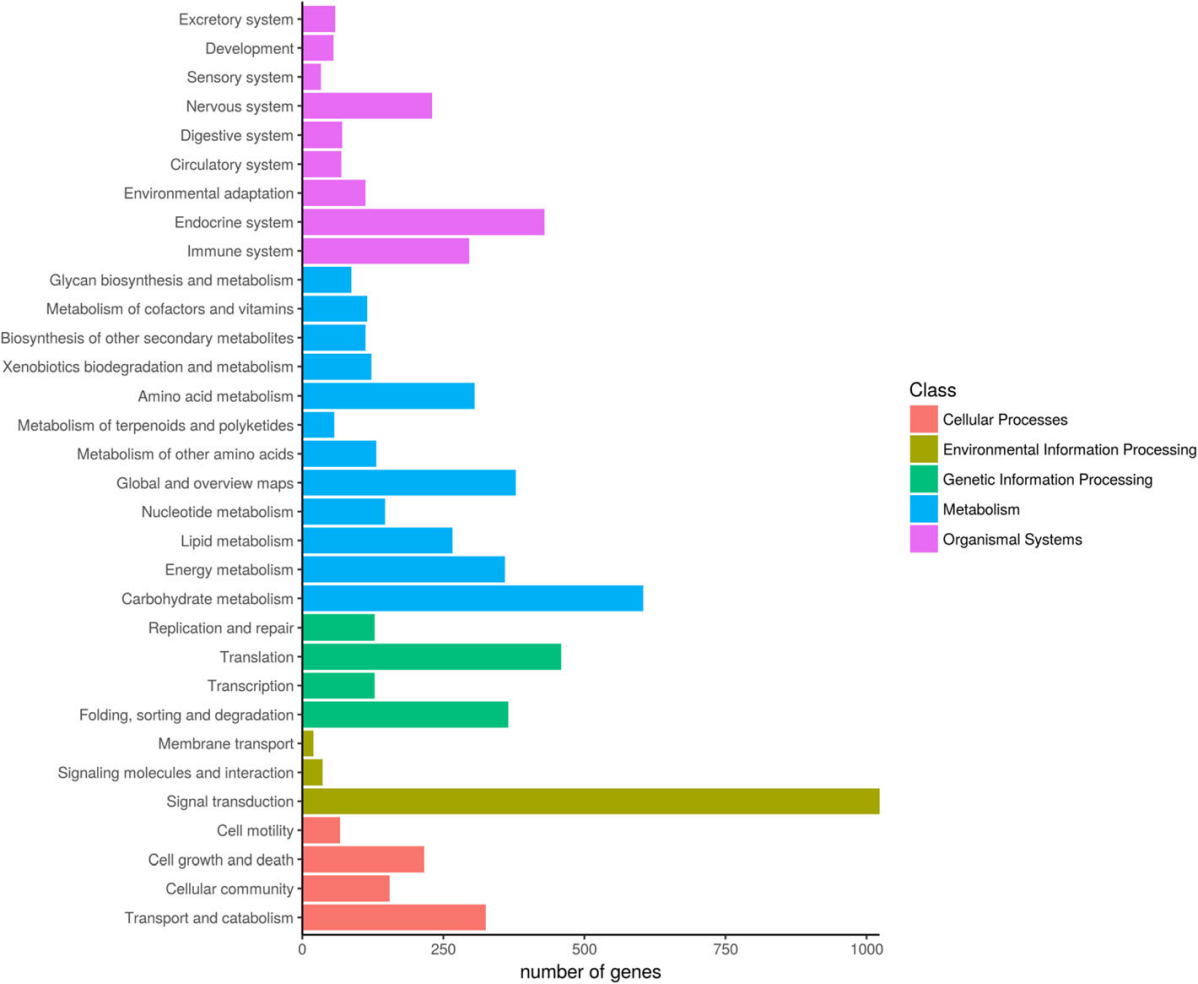
Functional classification and pathway assignment of assembled unigenes according to the KEGG database

**Fig. 3:**
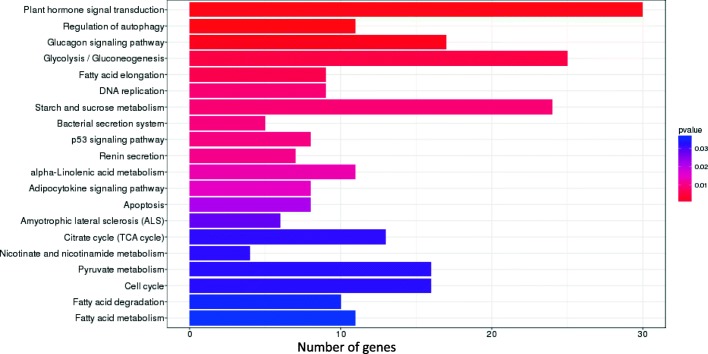
Analysis of the most enriched KEGG pathways corresponding to the DEGs between the blooming and withered flower samples of *Cymbidium faberi*

Unigenes of *C. faberi* were functionally categorized into three groups based on GO analysis, involved in biological process, cellular component and molecular function (Fig. 1). To identify the differentially expressed genes (DEGs) between these two developmental stages, the number of assembled genes was calculated with FPKM analysis. A total of 9409 DEGs were obtained, including 4581 up-regulated and 4828 down-regulated genes in blooming flower sample compared to the withered flower sample of *C. faberi*. Pathway enrichment analysis indicated that approximately 4209 unigenes participated in biological pathways identified by the Kyoto Encyclopedia of Genes and Genomes (KEGG). Based on the KEGG orthology (KO) and KEGG automatic annotation server (KAAS) methods, about 2821 pathways were screened out, with signal transduction, carbohydrate metabolism and endocrine system as the top three KEGG pathways (Fig. 2). A comparison of the KO enrichment results between S1 and S2 revealed 558 DEGs participating in 258 pathways. Among these, plant hormone signal transduction (ko 04075), glycolysis/gluconeogenesis (ko 00010), as well as starch and sucrose metabolism (ko 00500) were the top three pathways, followed by α-linolenic acid metabolism (ko 00592), pyruvate metabolism (ko 00620) and fatty acid degradation (ko 00071) (Fig. 3). These pathways included the conversion of α-linolenic acid to MeJA, which is one of the main VOC components of the flower scent of *C. faberi* [5]. The DEGs related to the MeJA metabolite in the KEGG database are summarized in Table 1. These were mainly related to α-linolenic acid metabolism (ko 00592) and fatty acid degradation (ko 00071). The transcripts of enzymes related to MeJA biosynthesis in the flowers of *C. faberi* were summarized in Table 2. These mainly included lipoxygenase (LOX: Unigene4670, Unigene54500, Unigene70202, Unigene49325, Unigene63843, Unigene20155, Unigene 54,798), allene oxide synthase (AOS: Unigene82057, Unigene20941), allene oxide cyclase (AOC: Unigene31970) and other enzymes catalyzed in β-oxidation of fatty acids. Most of the genes in the blooming flower sample showed high RNA abundance compared to the withered flower sample, among which the *CfJMT* gene (unigene 79,363), encoding jasmonic acid carboxyl methyltransferase, was particularly highly expressed in the blooming flower (Table 2).

**Table 1:** Summary of DEGs up- and down- regulated in the blooming flower sample compared to the withered flower sample of *Cymbidium faberi* related to MeJA metabolism according to the KEGG database

Pathway type	KEGG ID	# of DEGs	Up/Down (# of genes)	Unigene ID
α-linolenic acid metabolism	ko00592	11	10 Up	61,467; 20,941; 56,582; 3534; 69,403; 60,497; 28,969; 47,102; 51,380; 81,132
			1 down	29,317
Fatty acid degradation	ko00071	10	7 Up	56,582; 3534; 69,403; 67,030; 28,969; 47,102; 51,380
			3 Down	2730; 77,699; 35,801

**Table 2:** Summary of key enzymes and proteins related to MeJA metabolism in *Cymbidium faberi*

Gene ID	Log_2_Fold Change	*p*-value	Annotation
79,363	−2.97	8.53E-69	Jasmonic acid carboxyl methyltransferase
82,057	−5.54	1.19E-76	Allene oxide synthase
20,941	−2.55	1.18E-52	Allene oxide synthase
31,970	−4.28	7.11E-168	Allene oxide cyclase
4670	−5.99	1.67E-169	Lipoxygenase
54,500	−3.75	1.81E-36	Lipoxygenase
70,202	−3.4	0.009272813	Lipoxygenase
49,325	−1.4	9.38E-11	Lipoxygenase
63,843	−1.29	7.00E-12	Lipoxygenase
20,155	−6.01	5.54E-295	Lipoxygenase
54,798	6.93	3.14E-67	Lipoxygenase
56,582	−1.99	1.18E-42	Acyl-coenzyme A oxidase
4747	−1.3	0.001057461	Acyl-coenzyme A oxidase
28,969	−1.56	6.43E-23	Acyl-coenzyme A oxidase
60,497	−2.13	3.12E-28	12-oxophytodienoate reductase
47,102	−1.69	2.11E-12	Alcohol dehydrogenase
2730	4.09	4.43E-16	Aldehyde dehydrogenase
3534	−4.9	7.52E-36	Peroxisomal fatty acid β-oxidation multifunctional protein
69,403	−4.83	7.52E-36	Peroxisomal fatty acid β-oxidation multifunctional protein
29,317	3.26	1.07E-09	Triacylglycerol lipase SDP1
81,132	−6.05	4.39E-06	Anthranilate O-methyltransferase 3-like
67,030	−1.97	5.36E-33	Probable acyl-CoA dehydrogenase IBR3
77,699	3.92	3.21E-14	Long-chain acyl-CoA synthetase
35,801	3.83	2.46E-13	Long-chain acyl-CoA synthetase

Log_2_ Fold Change = Log_2_ (S2-RPKM/S1-RPKM)

### Identification of transcription factor families and validation of DEGs by qRT-PCR

**Fig. 4:**
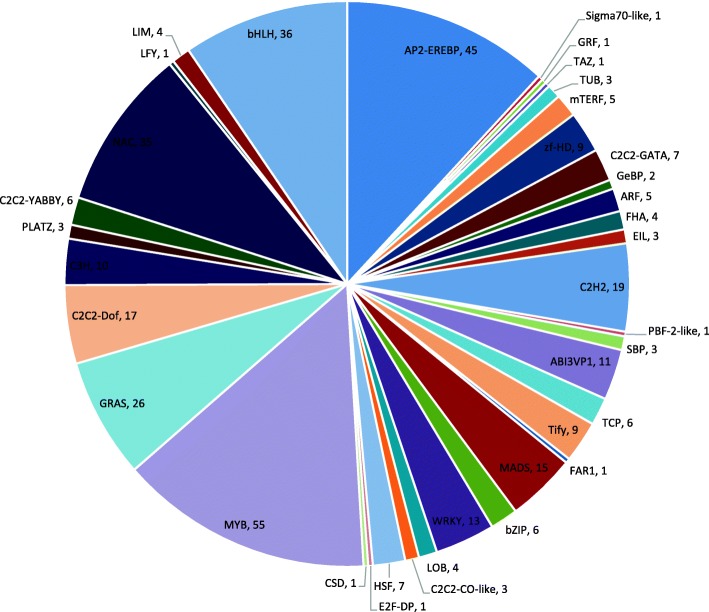
Distribution of differentially expressed transcription factors according to families

**Fig. 5:**
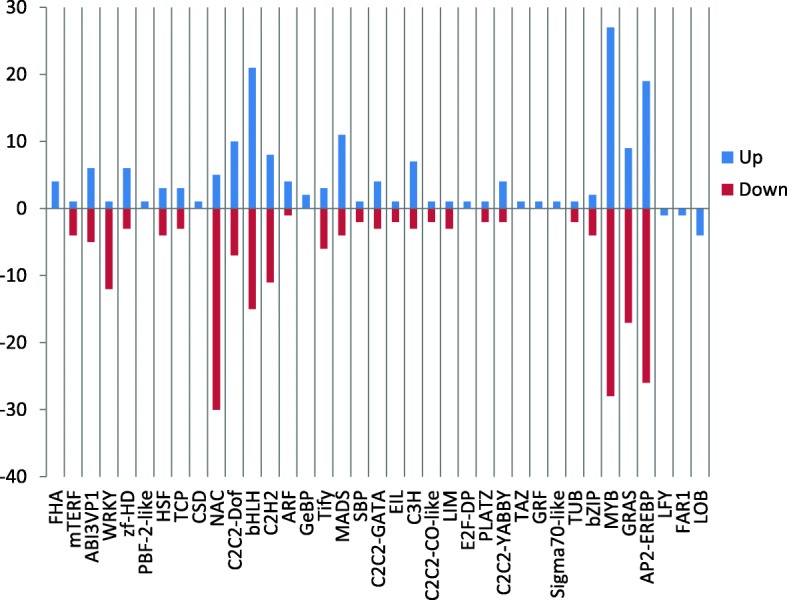
Up- and down-regulated TFs in the blooming flower sample compared to the withered flower sample of *Cymbidium faberi*

The main component of flower scent in *C. faberi* is MeJA, which is regulated by the crosstalk of many plant hormones [28]. It is necessary to identify the differentially expressed transcription factors (TFs) between S1 and S2 samples, which play an important role in the regulation of plant development. A total of 934 transcription factors were identified, belonging to 43 transcription factor families according to the PlantTFDB database, including the MYB family (129, 13.8%), AP2-EREBP (87, 9.3%) and bHLH (72, 7.7%) as the top 3 classes (Additional file 1: Figure S4). Among these, a total of 379 transcription factors identified as belonging to 37 transcription factor families were differentially expressed between the two samples. The top 10 groups belonged to the MYB, AP2-EREBP, bHLH, NAC, GRAS, C2H2, C2C2-Dof, MADS, WRKY and ABI3VP1 families (Fig. 4). A total of 172 TFs were up- and 207 were down-regulated in the blooming flower sample (Fig. 5).

**Fig. 6:**
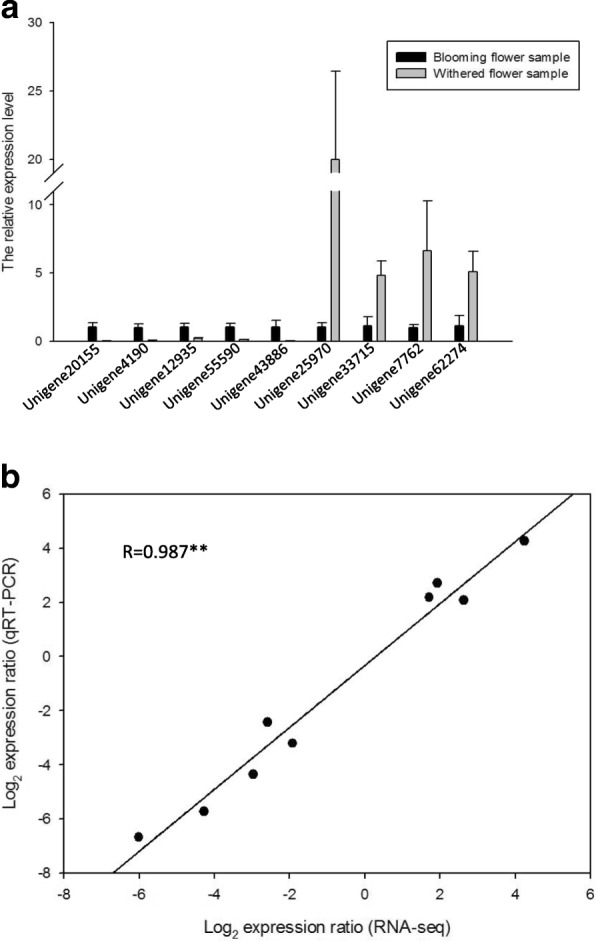
qRT-PCR validation of differential expressed unigenes. **a**. The expression of nine randomly selected unigenes was analyzed by qRT-PCR. The data represent the means and standard errors from three biological replicates. **b**. Correlation analysis of the gene expression ratios calculated from the qRT-PCR and RNA-seq data. The qRT-PCR log2 values (y-axis) were plotted against the RNA-seq log2 values (x-axis). The asterisks on the correlation coefficient (R) indicates the highly significant difference at *P* <0.01

To validate the results of the RNA-seq based DEG analysis, nine random DEGs were selected to perform qRT-PCR, including five up-regulated (Unigene 20,155, 4190, 12,935, 55,590 and 43,886) and four down-regulated unigenes (Unigene 25,970, 33,715, 7762 and 62,274) derived from the blooming flower sample. The primers were designed according to the assembled sequences of unigenes using Primer Premier 5.0 (Additional file 2: Table S1). The selected unigenes were successfully amplified and products were of the expected size, suggesting that the assembly work was reliable. The qRT-PCR result showed that the relative expression levels of the unigenes were consistent with the results of RNA-seq (Fig. 6). Hence, the unigenes that were up-regulated in the blooming flower sample showed higher transcript levels than the down-regulated unigenes, as expected.

### The full length coding region and promoter of the *CfJMT* gene were cloned and analyzed

A PCR product of about 1100 bp was obtained using the blooming flower cDNA as the template (Additional file 1: Figure S5). The sequencing result showed that the ORF of the *CfJMT* gene was 1125 bp in length, encoding 374 amino acids according to the ProtParam analysis. The calculated molecular mass of the encoded protein was about 41 kDa, with a theoretical pI of 5.05. The proportion of polar amino acids (72.89%) was much higher than that of nonpolar amino acids (27.11%), indicating a soluble protein. The N-terminus of CfJMT was found to contain an apparent chloroplast transit peptide signal before Arg^45^ according to ChloroP 1.1 analysis.

**Fig. 7:**
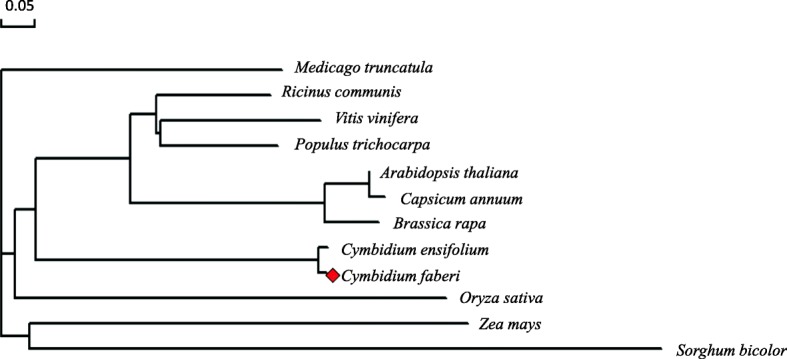
Homology analysis of selected plant JMT proteins

A total of 11 JMT protein sequences were downloaded from the NCBI database to construct a homology tree of CfJMT using DNAMAN (Fig. 7). CfJMT shared 98% homology with CeJMT, and the JMT proteins derived from monocots clustered together, suggesting that the function of JMT was highly conserved among closely related plant species.

In the first cycle of TAIL-PCR, a fragment of about 900 bp was obtained (Additional file 1: Figure S6a). The sequencing results revealed a fragment of about 180 bp overlapping with the ORF of *CfJMT*, including the ATG start codon. The second cycle of TAIL-PCR yielded another fragment with a length of about 700 bp (Additional file 1: Figure S6b). After sequencing and alignment, the promoter region spanning 1377 bp was successfully cloned. PlantCARE analysis predicted many cis-acting elements within the cloned sequence (Table 3), mainly including G-box and abiotic-responsive elements, suggesting that the expression of *CfJMT* is influenced by many environmental factors and controlled by a plethora of transcription factors.

**Table 3:** Cis-acting elements in the *CfJMT* promoter predicted by PlantCARE

Site name	Organism	Position	Strand
AAGAA-motif	*Avena sativa*	1205	–
AAGAA-motif	*Avena sativa*	1353	–
ABRE	*Arabidopsis thaliana*	547	+
ABRE	*Arabidopsis thaliana*	1493	–
ARE	*Zea mays*	1420	–
ARE	*Zea mays*	1459	–
AuxRR-core	*Nicotiana tabacum*	166	–
Box 4	*Petroselinum crispum*	1377	–
Box 4	*Petroselinum crispum*	1385	–
Box I	*Pisum sativum*	1123	+
Box III	*Pisum sativum*	301	+
Box-w1	*Petroselinum crispum*	1080	–
C-box	*Arabidopsis thaliana*	215	+
CGTCA-motif	*Hordeum vulgare*	394	+
EIRE	*Nicotiana tabacum*	1487	+
ERE	*Dianthus caryophyllus*	1122	+
G-BOX	*Antirrhinum majus*	547	–
G-BOX	*Pisum sativum*	1493	–
G-BOX	*Pisum sativum*	1088	–
G-BOX	*Daucus carota*	547	+
G-BOX	*Brassica napus*	1492	–
G-BOX	*Zea mays*	1088	–
G-BOX	*Arabidopsis thaliana*	1493	–
GARE-motif	*Brassica oleracea*	235	+
GC-motif	*Zea mays*	500	–
GT1-motif	*Avena sativa*	753	+
GT1-motif	*Arabidopsis thaliana*	1346	+
Gap-box	*Arabidopsis thaliana*	1440	–
Gap-box	*Arabidopsis thaliana*	1479	–
HSE	*Brassica oleracea*	818	–
HSE	*Brassica oleracea*	1349	–
HSE	*Brassica oleracea*	1304	+
I-box	*Flaveria trinervia*	1261	+
LTR	*Hordeum vulgare*	105	–
LTR	*Hordeum vulgare*	908	–
MBS	*Arabidopsis thaliana*	277	–
MBSI	*Petunia hybrida*	1339	+
O2-site	*Zea mays*	118	+
Skn-1 motif	*Oryza sativa*	395	+
Skn-1 motif	*Oryza sativa*	405	–
Sp1	*Zea mays*	312	+
TATCCAT/C-motif	*Oryza sativa*	208	–
TC-rich repeats	*Nicotiana tabacum*	934	–
TGACG-motif	*Hordeum vulgare*	394	–
TGGCA-motif	*Triticum aestivum*	352	+
W box	*Arabidopsis thaliana*	1080	–

### The *CfJMT* gene was highly expressed in flowers, especially at the blooming stage, but was almost undetectable in vegetative tissues

**Fig. 8:**
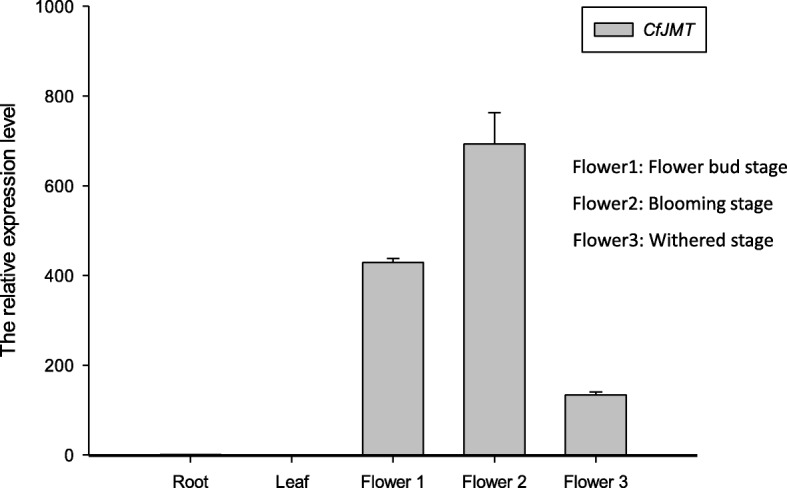
The spatio-temporal expression pattern of *CfJMT* gene in *Cymbidium faberi* as determined by qRT-PCR. Flower 1: flower bud stage; Flower 2: blooming stage; Flower 3: withered stage. The data represent the means and standard errors from three biological replicates

The spatio-temporal expression pattern of the *CfJMT* gene was analyzed in root, leaf and flowers from three stages of *C. faberi* by qRT-PCR, with the endogenous *β-actin* gene as the internal control. As can be seen in Fig. 8, the transcript of the *CfJMT* gene was almost undetectable in root and leaf tissues, while the relative expression level was up to 700-fold higher in flowers, especially during the blooming stage. Thus, the *CfJMT* gene may participate in flower morphogenesis in the blooming flower of *C. faberi*, influencing phenotypic traits such as coloration, flower development and fragrance biosynthesis, but not in vegetative growth.

### Constitutive ectopic expression of the *CfJMT* gene in tomato could not increase the volatile emission of MeJA, but the related biosynthetic genes were up-regulated following wounding

**Fig. 9:**
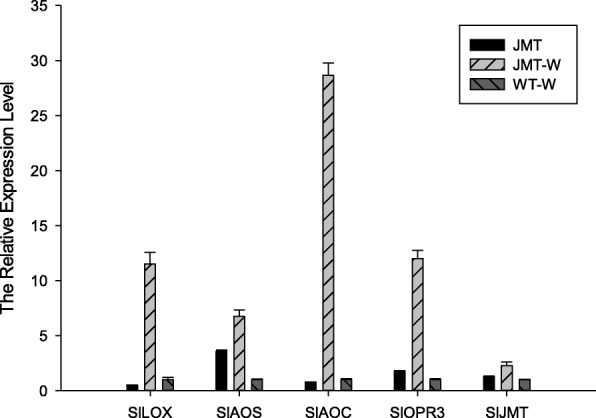
The transcription levels of genes related to MeJA biosynthesis in transgenic CfJMT-overexpressing and wild-type (WT) tomato plants that underwent mechanical wounding. The data represent the means and standard errors from three biological replicates. MeJA: methyl jasmonate

The *CfJMT* gene was successfully cloned into the pCambia1300 expression vector, which contains a CaMV 35S promoter and a *nos* terminator (Additional file 1: Figure S7), and was introduced into tomato plants for ectopic expression. About 20 independent transformants were obtained and grown in soil at 22 °C using a 16 h light and 8 h dark cycle. The positive transformants were preliminarily selected by PCR with an expected 438 bp *CfJMT* fragment (Additional file 1: Figure S8). The transformant with the highest RNA expression level was selected to analyze the components of its flower scent. However, MeJA was not found in tomato flower scent (data not shown). The same transformant was wounded to detect the influence of *CfJMT* on the plant response to external stimuli. After wounding, the transcription of genes related to MeJA biosynthesis was determined using qRT-PCR. The results showed that the transcription levels of *SlLOX*, *SlAOS*, *SlAOC*, *SlOPR3* and *SlJMT* in the transformant were increased 11.49-, 6.74-, 28.66-, 11.99- and 2.28-fold, respectively, compared to the analogously wounded wild-type control (Fig. 9).

## Discussion

Oriental orchids have the longest cultivation history, and retain great appreciative and economical value to this day [29]. Among them, *Cymbidium faberi* Rolfe is one of the most significant species with an elegant flower fragrance, but the biosynthesis mechanism of its flower scent remains unclear, which hinders the direct genetically informed breeding of this orchid. In this study, comparative transcriptome was applied to identify DEGs and metabolic pathways involved in the flower scent biosynthesis pathway of *C. faberi*. Based on the analysis and screening, a total of 9409 DEGs were obtained, 558 of which were found to participate in 258 metabolic pathways. The top pathways with the most DEGs are shown in Fig. 3. Most of them were found to be involved in hormonal signal transduction, which is a crucial factor for plant development and senescence, as was reported for *Rosa chinensis* [30]. MeJA, one of the main components in flower fragrance of *C. faberi*, is also one of the most important hormones in plants. In addition, ethylene (ET), salicylic acid (SA), abscisic acid (ABA) and other hormones also play important roles in the regulation of flower development between the blooming stage and withered stage of *C. faberi*.

**Fig. 10:**
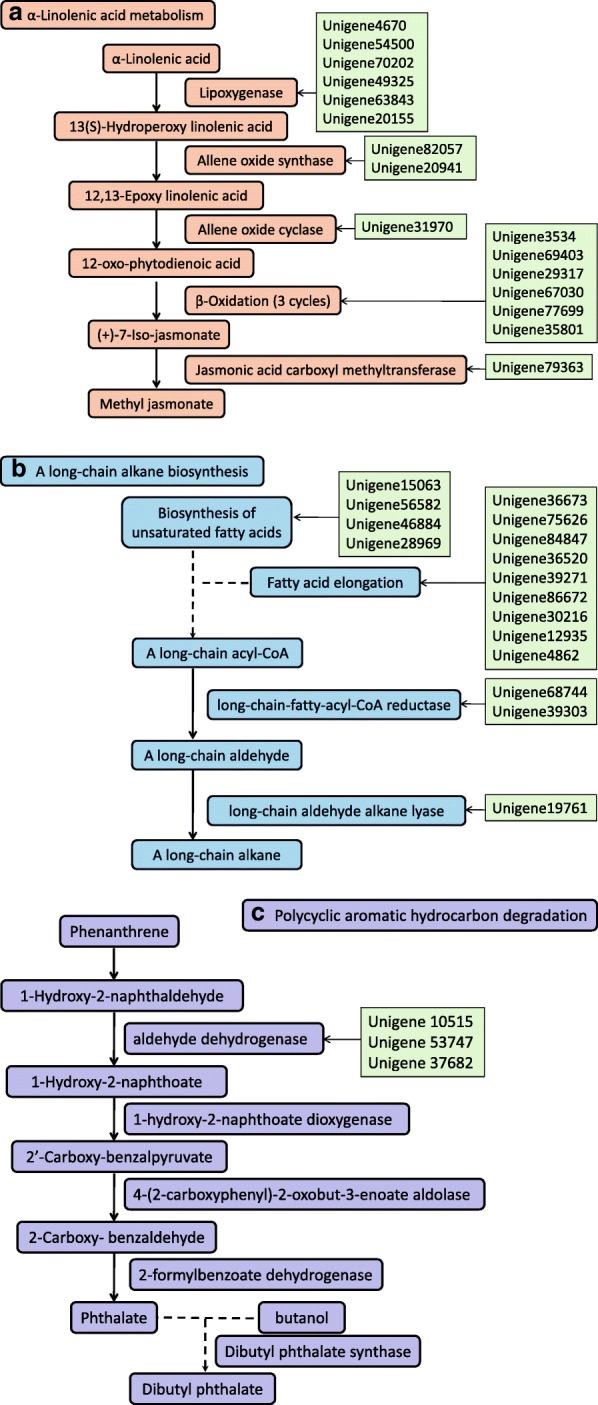
The main metabolic pathways and related unigenes in flower scent biosynthesis of *Cymbidium faberi* based on the KEGG analysis. **a**. α-linolenic acid metabolism pathway; **b**. A long-chain alkane biosynthetic pathway; **c**. Polycyclic aromatic hydrocarbon degradation pathway. The full lines represented the validated metabolic reactions and the dotted lines represented the predicted metabolic reactions

The main component of the flower scent of *C. faberi* is MeJA [5], which is a secondary product of α- linolenic acid metabolism (ko 00592), as listed in the top pathways. Additionally, the biosynthesis of JA also includes three cycles of β-oxidation, which is a part of fatty acid degradation pathway (ko 00071) (Table 1). The DEGs involved in the top pathways are summarized in Table 2 to illustrate the variation of their transcript levels, which also provides the direction for more extensive research on target pathways and genes. As one of the most important plant hormones, MeJA has attracted much attention in recent years. The biosynthetic pathway of MeJA has been elucidated in many model plants, and more research has been done on the regulation of the pathway and its crosstalk with other hormones [28]. The metabolic pathway of MeJA in *C. faberi* and the positions of related enzymes expressed by the corresponding unigenes are shown in Fig. 10a. The formation of MeJA started from the oxidation of α- linolenic acid via the lipoxygenase (mainly Unigene 4670), and then the 13(S)-Hydroperoxy linolenic acid was catalyzed by allene oxide synthase (Unigene82057 and Unigene20941) and allene oxide cyclase (Unigene31970) to form 12-oxo-phytodienoic acid (OPDA), which was transferred from chloroplasts to the peroxisomes, where three cycles of β-oxidation (Unigene3534, Unigene69403, Unigene29317, Unigene67030, Unigene77699 and Unigene35801) yielded jasmonic acid. The last step was methylated by jasmonic acid carboxyl methyltransferase (Unigene79363) to form the final product of MeJA.

The VOCs produced by plants are generally divided into three classes based on their biosynthetic origin, including the terpenoids, phenylpropanoids and fatty acid derivatives [31]. According to the GC/MS analysis, the top two compound classes in the flower scent of *C. faberi* were alkanes and esters [4]. The alkanes included 2,6,10-trimethyl-dodecane (C_15_H_32_), hexadecane (C_16_H_34_), heptadecane (C_17_H_36_), octadecane (C_18_H_38_) and 2,6,10,14-tetramethyl-hexadecane (C_20_H_42_). The main esters were MeJA (C_13_H_20_O_3_) and dibutyl phthalate (C_16_H_22_O_4_). The metabolic pathways of MeJA, alkanes and dibutyl phthalate, as well as the related unigenes are summarized in Fig. 10. Based on the KEGG pathway analysis these metabolites are produced through α-linolenic acid metabolism, a long-chain alkane biosynthetic pathway and polycyclic aromatic hydrocarbon degradation (PAH), respectively. The functions of most of the enzymes in α-linolenic acid metabolism and the long-chain alkane biosynthesis have been reported [7, 9, 32, 33]. However, limited research was done on the PAH degradation pathway [34]. The up- and down-regulated genes in MeJA biosynthesis of blooming flowers are summarized in Tables 1 and 2. In the long-chain alkane biosynthetic pathway, the transcription levels of several enzymes were up-regulated in the blooming stage, including long-chain-fatty-acyl-CoA reductase (Unigene 39,303) and long-chain aldehyde alkane lyase (Unigene 19,761). In the PAH degradation pathway, the aldehyde dehydrogenases (Unigene 10,515, Unigene 53,747 and Unigene 37,682) were up-regulated in the blooming flowers.

Among the differentially expressed transcription factors, the MYB and bHLH families were most highly represented, with 55 and 36 members in the list, respectively. It is well known that MeJA signal transduction is stringently regulated by MYC and MYC-like TFs in plants [35–39]. Jasmonate ZIM-domain (JAZ) proteins are targets of the E3 ligase SCF^COI1^, and MYC proteins is a target of JAZ proteins and a major regulator of JA signaling [40]. Four MYC proteins were found to be partially responsive to the JA signaling pathway in *Arabidopsis* [8, 41]. However, the *myc2myc3myc4* triple mutant still retained a partial response to JA signaling [42], which promoted the discovery of an ABA-inducible bHLH-type transcription factor, which was subsequently renamed as JA-associated MYC2-like (JAM) [43]. As JAM3 antagonistically acted on target genes of MYCs, it was recognized as a negative regulator of the JA signaling pathway in *Arabidopsis* [9, 44].

JMT, which was identified as one of the DEGs in the RNA-seq analysis, is the crucial enzyme for the formation of MeJA from JA [7]. In this study, the overexpression of *CfJMT* did not increase the content of MeJA among the VOCs, but the expression of related genes in MeJA biosynthesis upon wound treatment was changed in transgenic tomato plants, suggesting that *CfJMT* might influence the response of tomato to the external environment to some extent. However, the ectopic overexpression was not enough to increase the final titer of MeJA. It is possible that the amount of JA, the precursor of MeJA, limited the metabolic flux. By contrast, the overexpression of *AtJMT* in *Arabidopsis* led to a 3-fold increase of the MeJA without altering the JA content [7]. Similarly, when the *NTR1* gene from *Brassica campestris*, encoding jasmonic acid carboxyl methyltransferase, was overexpressed in soybean, it resulted in 2- to 2.5- fold higher levels of MeJA than in the control [11]. Notably, the recombinant *CeJMT* derived from *Cymbidium ensifolium* was able to specifically catalyze the conversion of JA into the corresponding methyl ester MeJA [21]. However, the expression of a functional *FvJMT* was negatively correlated with strawberry fruit development [13]. Similarly, the recently described SAMT enzyme (S-adenosyl-L-methionine: salicylic acid carboxyl methyltransferase) responsible for the biosynthesis of methyl salicylate and methyl benzoate is not involved in floral scent production in snapdragon flowers [45].

A number of conserved cis-acting elements were predicted in the *CfJMT* promoter sequence (Table 3). Some G-box elements might be targets for MYC transcription factors [46]. In the comparative transcriptome analysis, some candidate MYC transcription factors were identified as potentially participating in the biosynthesis of MeJA, as well as the flower scent of *C. faberi*.

*CfJMT* was almost uniquely expressed in flowers, and was barely detectable in the vegetative organs, which suggested that this enzyme is closely related with flower development in *C. faberi*. The regulation of gene expression is most likely mediated by changes of the promoter activity. In this study, we could not find any mutations in the *CfJMT* promoter sequence, and further research will be required to elucidate how the conserved cis-elements are recognized and regulated by the corresponding transcription factors.

## Conclusions

In this study, comparative transcriptome analysis of differentially expressed genes between blooming and withered flowers of *C. faberi* revealed DEGs and metabolic pathways that are crucial for flower development and the biosynthesis of flower scent, providing a basis for studying the corresponding molecular mechanisms. Some of the identified transcription factors and enzymes appear to be closely linked to flower scent biosynthesis. Since the focus of the study was on the important genes contributing to the formation of MeJA, the *CfJMT* gene was cloned and its function was confirmed. These results will hopefully promote more intensive research on the formation and regulation of floral scent in *C. faberi*, which also provide new insights for molecular breeding, and may help preserve wild orchid resources.

## Methods

### Collection of plant materials and RNA extraction

The wild *C. faberi* was transplanted from the mountain of Dangyang (30°55′25″N, 111°51′24″E), Hubei province, China in 2011, and preserved at the greenhouse of Wuhan University of Bioengineering. After the flower buds fully expanded in 1 day (flower bud stage), the flowers started to emit strong fragrance (blooming stage), after which they kept almost the same morphology and lasted about 10 days. Then the flowers gradually lost the fragrance and the sepals become curled (withered stage). About one week later, the flowers abscised from the rachis and were dead. The freshly blooming flowers and withered flowers were collected respectively at about 20 days before senescence (dbs) and 7 dbs at 12:00 AM, when *C. faberi* typically has the strongest scent [21]. The fresh flowers were detected the VOCs in flower fragrance following the details in Zhou et al. [4]. The corresponding samples were collected for RNA-seq. Three flowers were isolated from the top of the rachis derived from three individuals at the same stage as one sample, and immediately frozen in liquid nitrogen. Three replicates were prepared for each sample. The total RNAs was extracted using the RNAiso Plus kit (Takara, China), following the manufacturer’s instructions. The quality of the RNA was determined using a Nano Drop 2000 instrument (Thermo Fisher Scientific, USA) and Bioanalyzer 2100 (Agilent).

### Transcriptome sequencing, de novo assembly and functional annotation

A total of 10 μg of RNA was prepared to synthesize cDNA libraries of the blooming flower and withered flower. The cDNA was synthesized and purified using NEBNext Ultra RNA library Prep Kit for Illumina and Poly (A) mRNA Magnetic Isolation Module (NEB). A total of 6 cDNA libraries (3 for the blooming stage and 3 for the withered stage) were finally sequenced using an Illumina HiSeq2500 platform, and paired-end reads were generated. The clean reads were obtained by removing reads containing adaptors or poly(A) and low-quality reads from the raw data. The remaining high-quality reads were de novo assembled into candidate unigenes using the Trinity software (r20140717). Assembled unigenes were annotated according to seven public databases, including UniProt, Nr, KEGG, KEGG Pathway, Gene Ontology, COG and Pfam by blast (2.2.28+) and HMMER (3.1). The reads were counted numbers and mapped to assembled transcripts by RSEM (1.2.25), after which the FPKM (using fragments per kilobase of a transcript per million mapped reads; false discovery rate ≤ 0.05 and |log_2_ratio| ≥ 1) of each gene was calculated based on the length of the gene and the reads count mapped to this gene. Differentially expressed genes with the threshold of FDR ≤ 0.05 and |log_2_FoldChange| > 1 were screened using DEseq2 (1.8.2). Nine random genes were selected to verify the result of RNA sequencing by qRT-PCR. Primers were designed using Primer Premier 5 software (Additional file 2: Table S1). Real-time PCR was conducted using ABI7500 (Thermo Fisher Scientific, Germany). The same cDNA that was used for RNA-seq served as the template. The reagents were mixed in a 10 μL system containing 5 μL 2 × SYBR Premix (Takara, China), and 0.25 μL upstream and downstream primers each (10 μM). The PCR procedure followed the default two-step settings: 95 °C for 10 min, followed by two steps of 95 °C for 15 s and 60 °C for 1 min. The fluorescent signal of the SYBR green dye was measured and the PCR procedure used the default settings as reference. The relative expression value was calculated using the2^- △△CT^ (Livak) method, △△C_T_ = (C_T_, gene- C_T_, actin) treatment- (C_T_, gene- C_T_, actin) control. The dissociation curves were used to detect primer dimers and other non-specific by-products. Samples were analyzed in biological triplicates.

### Cloning the coding sequence and promoter of the *JMT* gene of *C. faberi*

Total RNA was extracted from blooming flowers of *C. faberi* following the same protocol as for sample preparation in RNA-seq and the full length of *CfJMT* was cloned with the primers of JMT-F1 (GCTCTAGAATGGATGTCAAGCAAG, *Xba*I restriction site underlined) and JMT-R1 (CGGGATCCTTAAAGAGCCTTTCTAGGG, *BamH*I restriction site underlined). PCR procedures were as following: 94 °C for 5 min, and 32 cycles of 94 °C for 30 s, 53 °C for 45 s, and 72 °C for 1 min, 72 °C for 10 min and hold for 16 °C.

Genomic DNA with a concentration ~ 1600 ng/μL was isolated from young leaves of *C. faberi* using the CTAB protocol and was diluted 50-fold to yield the PCR template. A modified protocol of TAIL-PCR was applied to amplify the promoter of *CfJMT* as described previously [47]. Pre-amplification reactions (25 μL) were prepared, each containing 2.5 μL of PCR buffer (10×), 2 μL of dNTPs (10 mM each), 2 μL of GSP primer (10 μM), 1 μL of AD primer (10 μM), 0.1 μL of LA Taq (5 U/μL), 12.4 μL of ddH_2_O and 20~30 ng of template DNA. Primary TAIL-PCR reactions (25 μL) contained 2.0 μL of PCR buffer (10×), 4 μL of dNTPs (10 mM each), 2 μL of GSP primer (10 μM), 1 μL of AD primer (10 μM), 0.1 μL of LA Taq (5 U/μL), 12.4 μL of ddH_2_O and 3 μL of 50-fold diluted pre-amplified product. Each secondary 25 μL TAIL-PCR contained 2.0 μL of PCR buffer (10×), 4 μL of dNTPs (10 mM each), 2 μL of GSP primer (10 μM), 1 μL of AD primer (10 μM), 0.1 μL of LA Taq (5 U/μL), 12.4 μL of ddH_2_O and 3 μL of 10-fold diluted primary TAIL-PCR product.

To obtain the full length of the *CfJMT* promoter, two cycles of TAIL-PCR were applied. For cycle 1, amplifying the region close to the translational start codon, the three gene-specific primers SP1, SP2 and SP3 were designed based on the coding sequence of *CfJMT* (Additional file 2: Table S2). This step also included the universal primers AD1 to AD8, respectively, combined with the SP primers. According to the sequence of the cycle 1 product, another three primers for cycle 2 were designed and designated as 2SP1, 2SP2 and 2SP3 (Additional file 2: Table S2). For assembling the sequences of the final products of cycle 1 and cycle 2, another primer, 3SP, was designed to amplify the full length of the *CfJMT* promoter with SP2. The PCR conditions were 94 °C, 3 min; followed by 35 cycles at 94 °C, 30 s, 56 °C, 30 s, 72 °C, 2 min; and a final extension at 72 °C for 10 min.

### Bioinformatic analysis of the *CfJMT* gene

The *CfJMT* coding region was sequenced by Sangon Biotech (Shanghai, China). The sequence was analyzed using ProtParam to evaluate the molecular weight and isoelectric point of CfJMT protein (http://web.expasy.org/protparam/). The expected location of CfJMT was analyzed using ChloroP 1.1 software. The homologous JMT sequences from other species were downloaded from the NCBI GeneBank and the homology tree was constructed using DNAMAN software with the maximum likelihood algorithm. The sequences were derived from *Medicago truncatula* (XM_013610683), *Vitis vinifera* (XM_002281552), *Populus trichocarpa* (KC894590), *Brassica rapa* (XM_009151199), *Capsicum annuum* (DQ222856), *Arabidopsis thaliana* (NM_101820), *Ricinus communis* (XM_002529590), *Sorghum bicolor* (XM_002438267), *Oryza sativa* (EU146297.1), *Zea mays* (NP_001141374) and *Cymbidium ensifolium* (JQ360571).

The TAIL-PCR products of the promoter sequence of the *CfJMT* gene were sequenced by Sangon Biotech (Shanghai, China) and then assembled using ContigExpress software. PLACE software (http://bioinformatics.psb.ugent.be/webtools/plantcare/html/) was used to predict the cis-acting elements and DNA-binding regions within the *CfJMT* promoter sequence.

### Analysis of the spatio-temporal expression pattern of *CfJMT* in *C. faberi* by qRT-PCR

The expression pattern of the *CfJMT* gene was determined by qRT-PCR by analyzing tissue samples from roots, leaves, and flowers of *C. faberi*. The flower samples were separated into three stages: flower bud stage, blooming stage and withered stage. The fresh flowers were collected at about 22 days before senescence (dbs) (flower bud stage), 20 dbs (blooming stage) and 7 dbs (withered stage). Total RNAs were extracted following the same protocol as for sample preparation in RNA-seq. The cDNAs were synthesized using the PrimeScript II High Fidelity RT-PCR Kit (Takara, China), according to the manufacturer’s instructions. The gene-specific primers for *CfJMT* were qJMT-F1 (ATCCAAAGCATTATTGCGTCCCT) and qJMT-R1 (CACCAGCAGTGCATTCTTTCCAG). The intrinsic *actin* gene was used as the internal control, with the primers qActin-F (TTTATGAGGGTTATGCGCTTCC) and qActin-R (AACTACTGCAGAACGGGAAAT).

### Ectopic expression of the *CfJMT* gene in tomato plants

The vector for the heterologous expression of the *CfJMT* gene was constructed in accordance with previous research [4]. The resulting recombinant plasmid pC35ST-CfJMT was used to transform tomato plants as described previously [48]. The transformants were screened for positive seedlings by PCR and qRT-PCR. The primer pairs for PCR were JMT-F2 (5’-AGGAAGCGGCGATACAAG-3′) and JMT-R2 (5’-CGTGGCACCTGAGAAAGC-3′). The relative expression levels of genes involved in MeJA biosynthesis in tomato were determined using qRT-PCR to reflect the influence of *CfJMT* on MeJA biosynthesis in tomato. The primers for the qRT-PCR were the same as previous study [4]. After flowering, the fresh flower was collected to detect the contents of flower fragrance via GC-MS following the standard procedures [49].

### Wounding assay of transgenic tomato plants

The tomato transformants were cut with a blade to simulate mechanical wounding. After treatment for 2 h, the wounded leaves were collected to determine the relative expression levels of genes involved in MeJA biosynthesis in tomato, and compare it with that of analogously treated wild-type plants.

### Statistical analysis

Data on the relative expression levels of genes as determined by qRT-PCR were presented as the means ± SE from n = XY parallel experiments, and were subjected to analysis of variance using SigmaPlot 12.5 software.

## Additional files

Additional file 1: **Figure S1.** Changes in main components of flower fragrance during blooming stage and withered stage of *Cymbidium faberi*. n.d = not detected. **Figure S2.** The E-value distribution of assembled unigenes according to Nr database. **Figure S3.** The species distribution of assembled unigenes according to Nr database. **Figure S4.** Distribution of transcription factor families based on Pfam analysis. **Figure S5.** PCR amplification of the *CfJMT* gene from *Cymbidium faberi*. M: DL 5000 DNA Marker; lane 1: PCR product. **Figure S6.** Isolation of the *CfJMT* promoter from *Cymbidium faberi* by TAIL-PCR. a. Amplification cycle 1 used primer pairs comprising SP3 and AD universal primers from AD1 to AD8, respectively. b. Amplification cycle 2 used primer pairscomprising2SP3 and AD universal primers from AD1 to AD8, respectively. M: DL 2000 DNA Marker; Lane 1-8: AD universal primers from AD1 to AD8. The red arrows indicate the target amplification products based on the sequencing results. **Figure S7.** Vector map of pC35ST-*Cf*JMT with restriction sites and related elements. **Figure S8.** PCR analysis of the positive tomato transformants. M: DL 2000 DNA Marker; lane 1-6: PCR amplification of *CfJMT* fragments derived from the genomic DNA of independent tomato transformants; lane 7: PCR amplification of *Cf*JMT fragments derived from the genomic DNA of wild-type tomato plants. (PPTX 237 kb)

Additional file 2: **Table S1.** Primers derived from unigene sequences used for the verification of the RNA-seq results via qRT-PCR. **Table S2.** Primers used for TAIL-PCR. (DOCX 15 kb)

## Abbreviations

AOC: Allene oxide cyclase
AOS: Allene oxide synthase
DEGs: Differentially expressed genes
FPKM: Fragments per kilobase of a transcript per million mapped reads
GO: Gene ontology
JA: Jasmonic acid
JAM: JA-associated MYC2-like
JAZ: Jasmonate ZIM-domain
JMT: Jasmonic acid carboxyl methyltransferase
KAAS: KEGG automatic annotation server
KEGG: Kyoto Encyclopedia of Genes and Genomes
KO: KEGG orthology
LOX: Lipoxygenase
MeJA: Methyl jasmonate
OPDA: 12-oxo-phytodienoic acid
OPR3: OPDA reductase 3
PAH: Polycyclic aromatic hydrocarbon
SAMT: S-adenosyl-L-methionine: salicylic acid carboxyl methyltransferase
VOC: Volatile organic compounds
