# Supplementary material for: Transcriptomic profiling of the flower scent biosynthesis pathway of Cymbidium faberi Rolfe and functional characterization of its jasmonic acid carboxyl methyltransferase gene
Source: BMC Genomics. 2019 Feb 11;20:125. doi: 10.1186/s12864-019-5501-z (PMC6371524; doi:10.1186/s12864-019-5501-z)
Supplement: Supplementary file 1 — Figure S1. Changes in main components of flower fragrance during blooming stage and withered stage of Cymbidium faberi. n.d = not detected. Figure S2. The E-value distribution of assembled unigenes according to Nr database. Figure S3. The species distribution of assembled unigenes according to Nr database. Figure S4. Distribution of transcription factor families based on Pfam analysis. Figure S5. PCR amplification of the CfJMT gene from Cymbidium faberi. M: DL 5000 DNA Marker; lane 1: PCR product. Figure S6. Isolation of the CfJMT promoter from Cymbidium faberi by TAIL-PCR. a. Amplification cycle 1 used primer pairs comprising SP3 and AD universal primers from AD1 to AD8, respectively. b. Amplification cycle 2 used primer pairscomprising2SP3 and AD universal primers from AD1 to AD8, respectively. M: DL 2000 DNA Marker; Lane 1-8: AD universal primers from AD1 to AD8. The red arrows indicate the target amplification products based on the sequencing results. Figure S7. Vector map of pC35ST-CfJMT with restriction sites and related elements. Figure S8. PCR analysis of the positive tomato transformants. M: DL 2000 DNA Marker; lane 1-6: PCR amplification of CfJMT fragments derived from the genomic DNA of independent tomato transformants; lane 7: PCR amplification of CfJMT fragments derived from the genomic DNA of wild-type tomato plants. (PPTX 237 kb) [file 12864_2019_5501_MOESM1_ESM.pptx]

## Slide 1
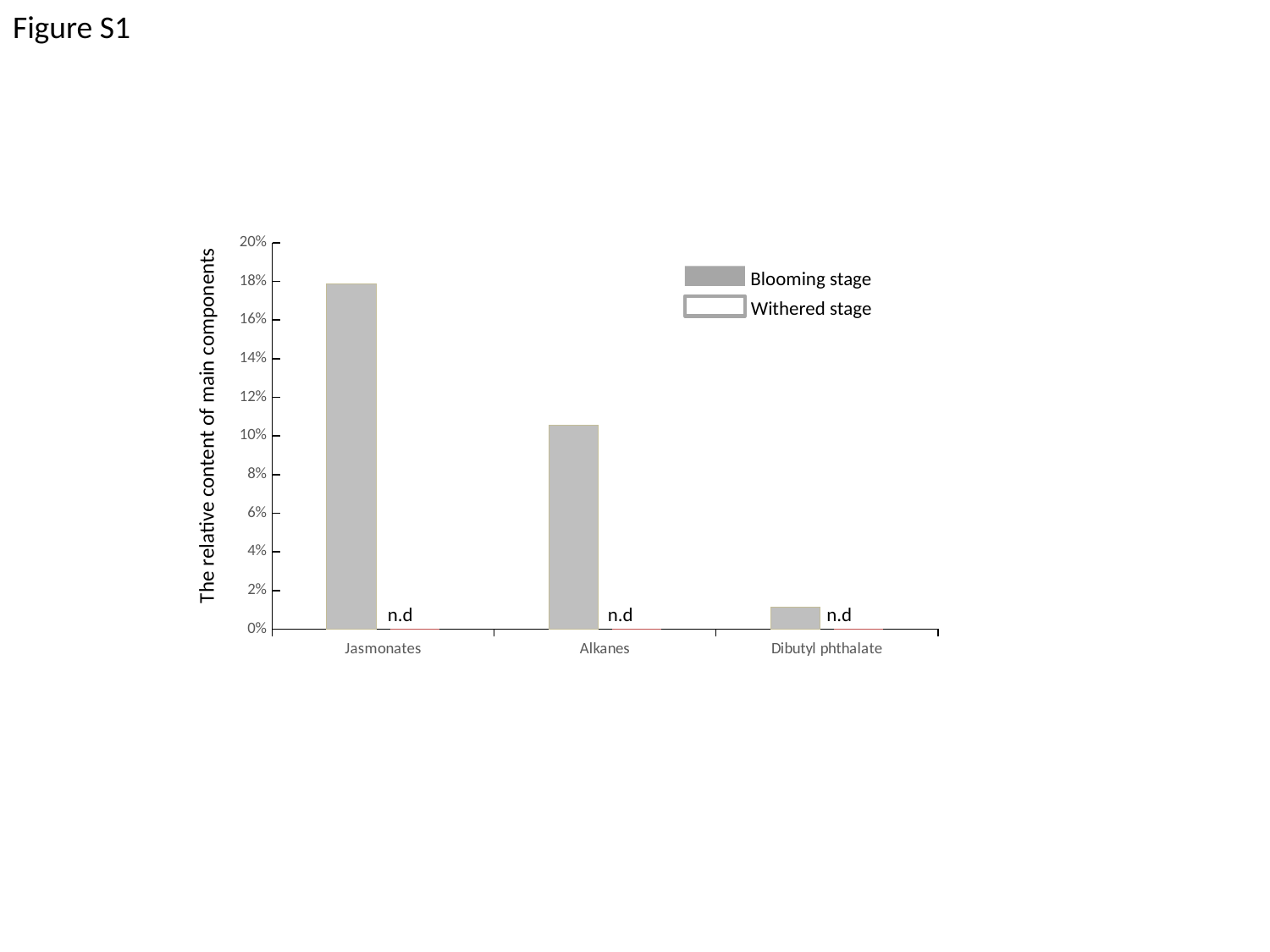

Figure S1
### Chart
| Category | Blooming stage | Withered stage |
|---|---|---|
| Jasmonates | 0.17870000000000003 | 0.00010000000000000002 |
| Alkanes | 0.10550000000000001 | 0.00010000000000000002 |
| Dibutyl phthalate | 0.011500000000000003 | 0.00010000000000000002 |Blooming stage
Withered stage
n.d
n.d
n.d
The relative content of main components

## Slide 2
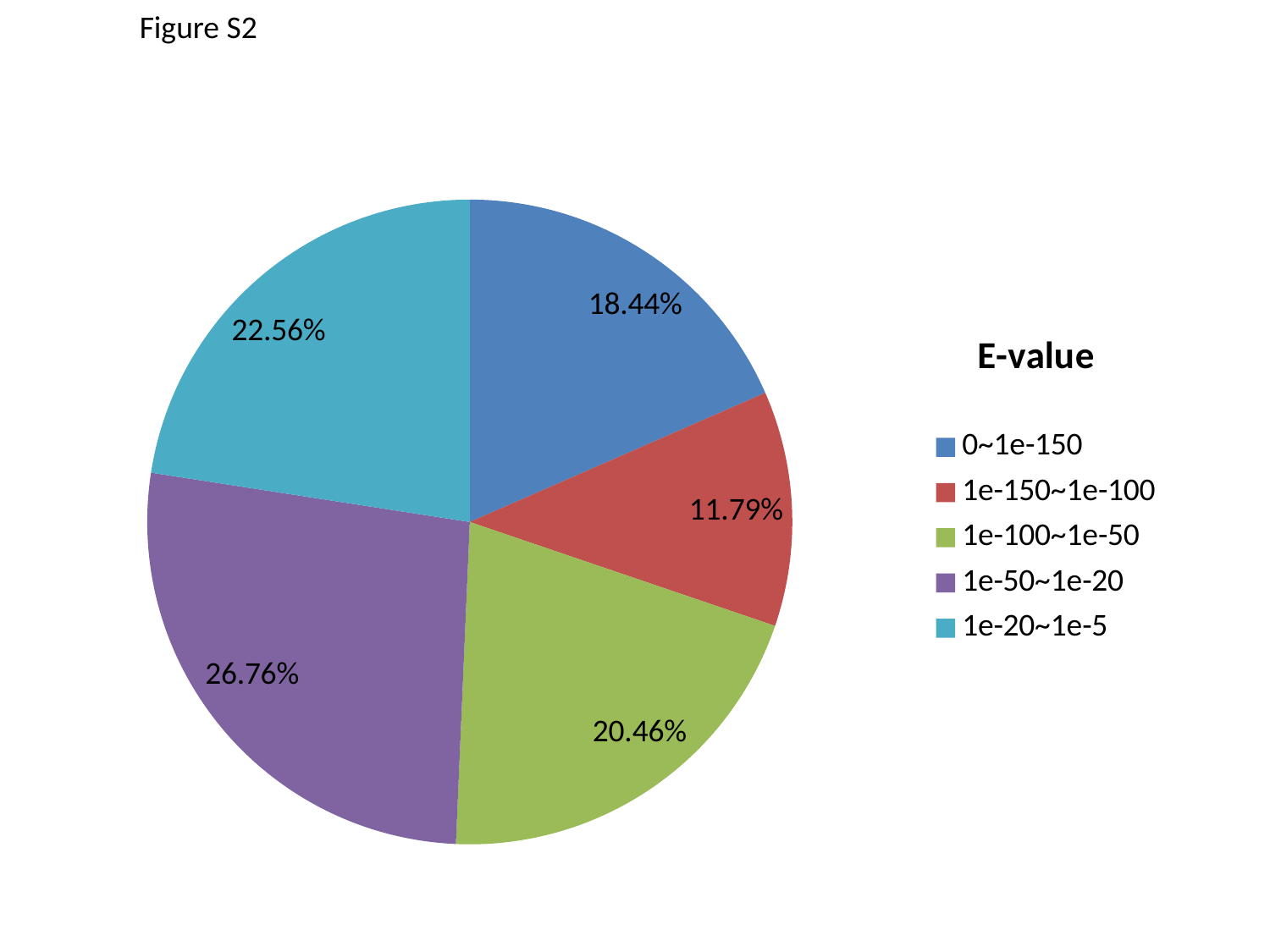

Figure S2
### Chart:
| Category | E-value |
|---|---|
| 0~1e-150 | 0.18440000000000029 |
| 1e-150~1e-100 | 0.1179 |
| 1e-100~1e-50 | 0.2046 |
| 1e-50~1e-20 | 0.2676 |
| 1e-20~1e-5 | 0.2256 |

## Slide 3
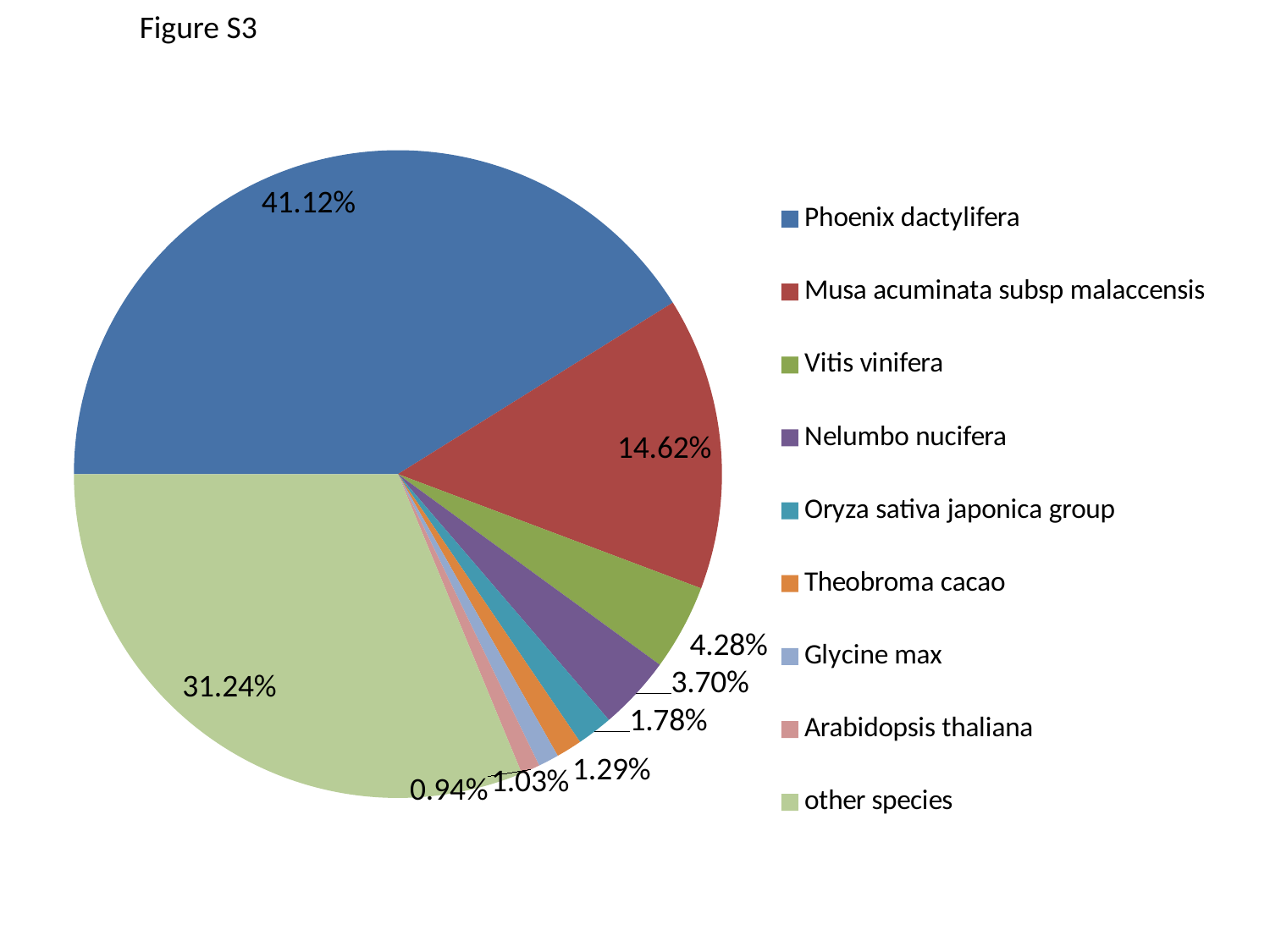

Figure S3
### Chart
| Category | Species |
|---|---|
| Phoenix dactylifera | 0.4112 |
| Musa acuminata subsp malaccensis | 0.14620000000000022 |
| Vitis vinifera | 0.04280000000000003 |
| Nelumbo nucifera | 0.037 |
| Oryza sativa japonica group | 0.017800000000000003 |
| Theobroma cacao | 0.012900000000000003 |
| Glycine max | 0.010300000000000005 |
| Arabidopsis thaliana | 0.009400000000000014 |
| other species | 0.3124000000000005 |

## Slide 4
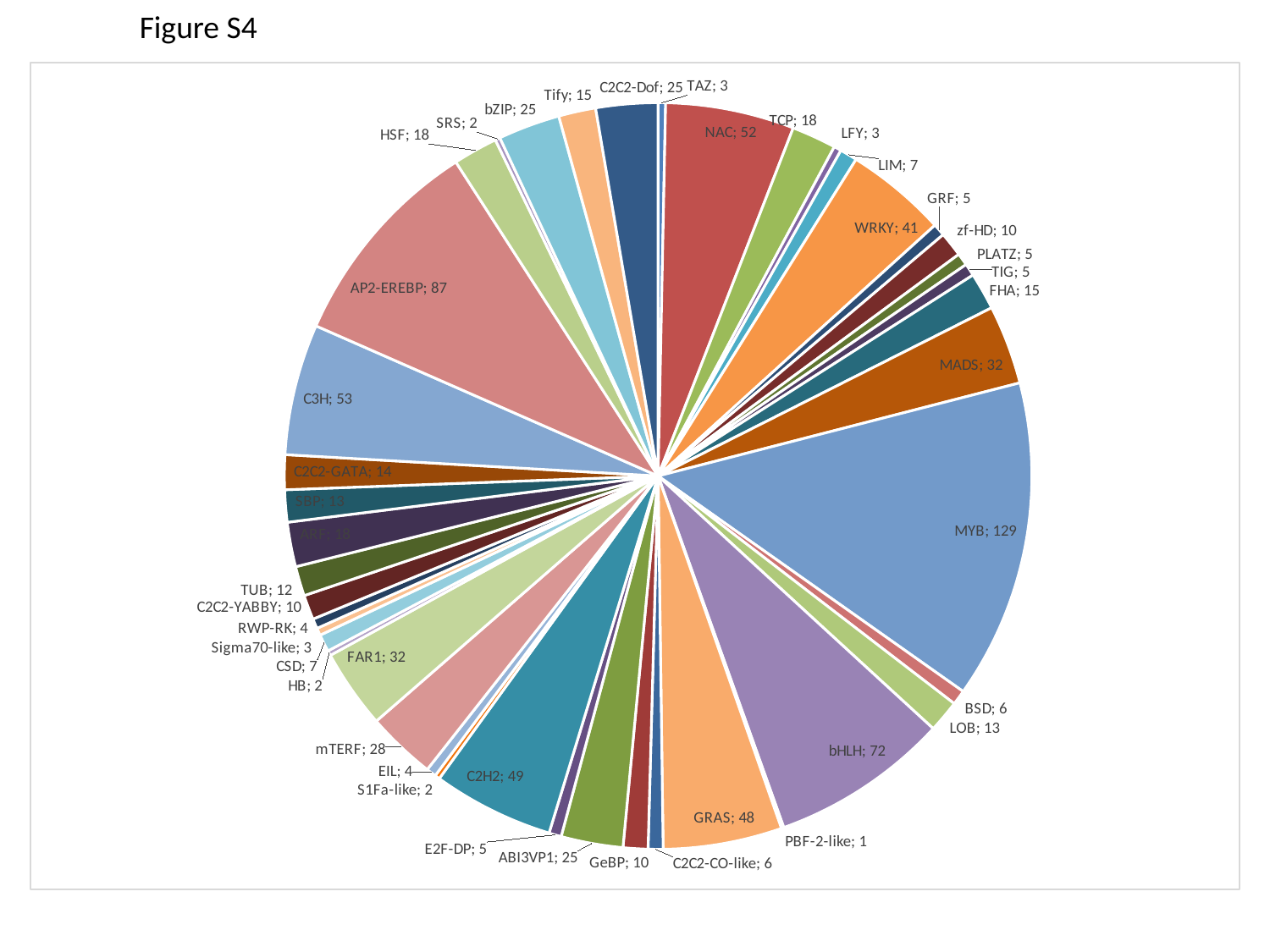

Figure S4
### Chart
| Category | |
|---|---|
| TAZ | 3.0 |
| NAC | 52.0 |
| TCP | 18.0 |
| LFY | 3.0 |
| LIM | 7.0 |
| WRKY | 41.0 |
| GRF | 5.0 |
| zf-HD | 10.0 |
| PLATZ | 5.0 |
| TIG | 5.0 |
| FHA | 15.0 |
| MADS | 32.0 |
| MYB | 129.0 |
| BSD | 6.0 |
| LOB | 13.0 |
| bHLH | 72.0 |
| PBF-2-like | 1.0 |
| GRAS | 48.0 |
| C2C2-CO-like | 6.0 |
| GeBP | 10.0 |
| ABI3VP1 | 25.0 |
| E2F-DP | 5.0 |
| C2H2 | 49.0 |
| S1Fa-like | 2.0 |
| EIL | 4.0 |
| mTERF | 28.0 |
| FAR1 | 32.0 |
| HB | 2.0 |
| CSD | 7.0 |
| Sigma70-like | 3.0 |
| RWP-RK | 4.0 |
| C2C2-YABBY | 10.0 |
| TUB | 12.0 |
| ARF | 18.0 |
| SBP | 13.0 |
| C2C2-GATA | 14.0 |
| C3H | 53.0 |
| AP2-EREBP | 87.0 |
| HSF | 18.0 |
| SRS | 2.0 |
| bZIP | 25.0 |
| Tify | 15.0 |
| C2C2-Dof | 25.0 |

## Slide 5
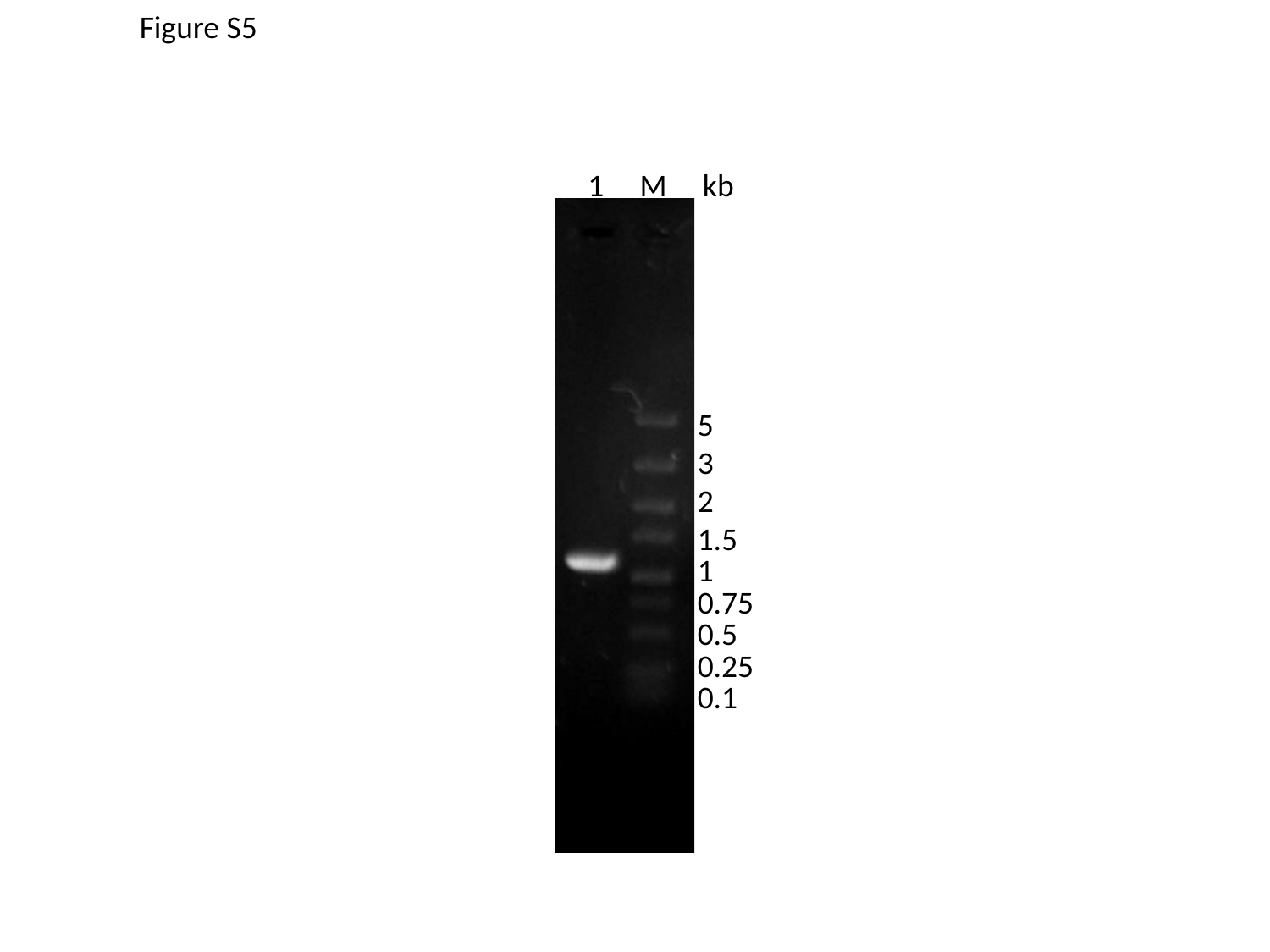

Figure S5
1 M kb
5
3
2
1.5
1
0.75
0.5
0.25
0.1

## Slide 6
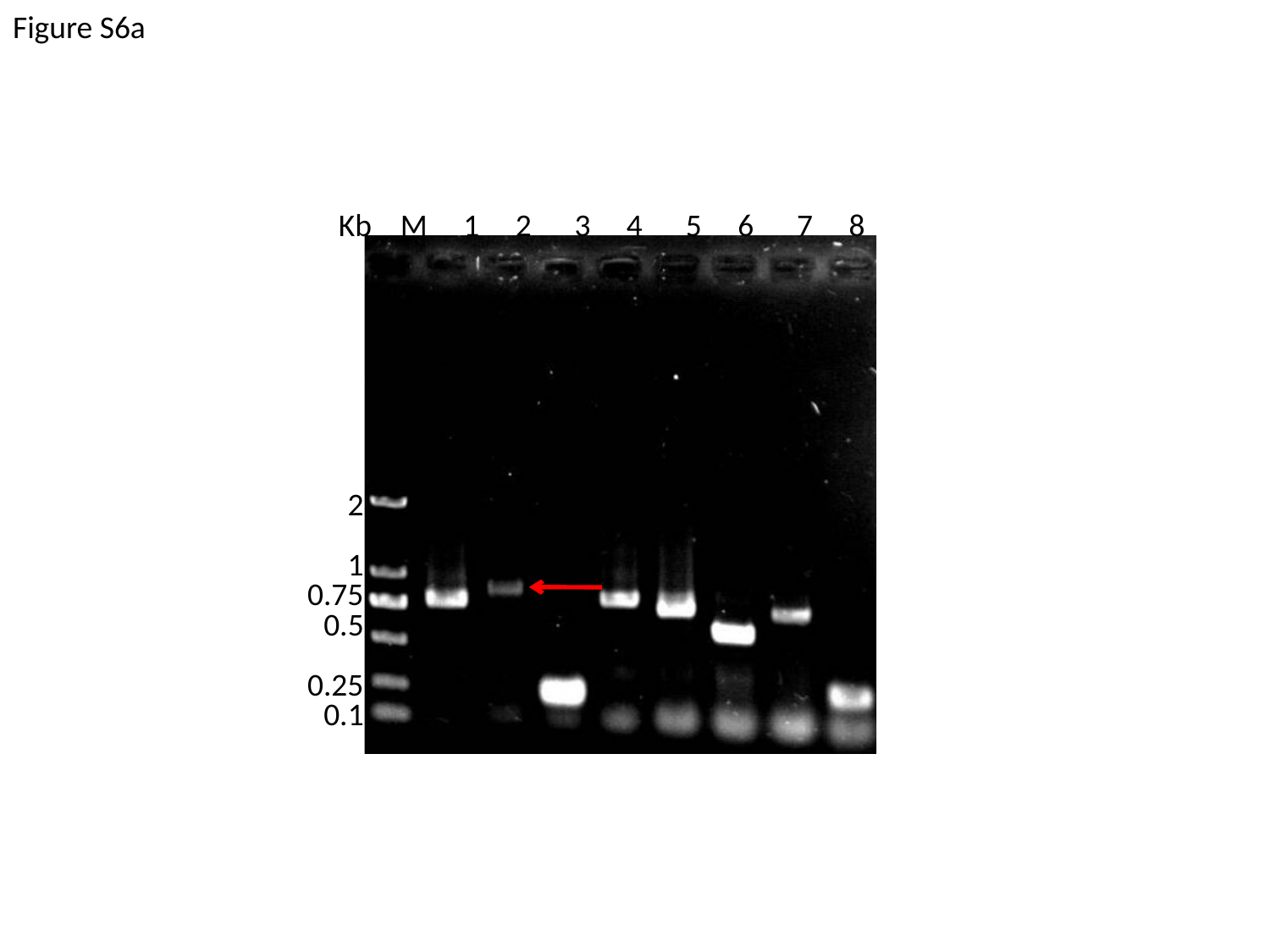

Figure S6a
Kb M 1 2 3 4 5 6 7 8
2
1
0.75
0.5
0.25
0.1

## Slide 7
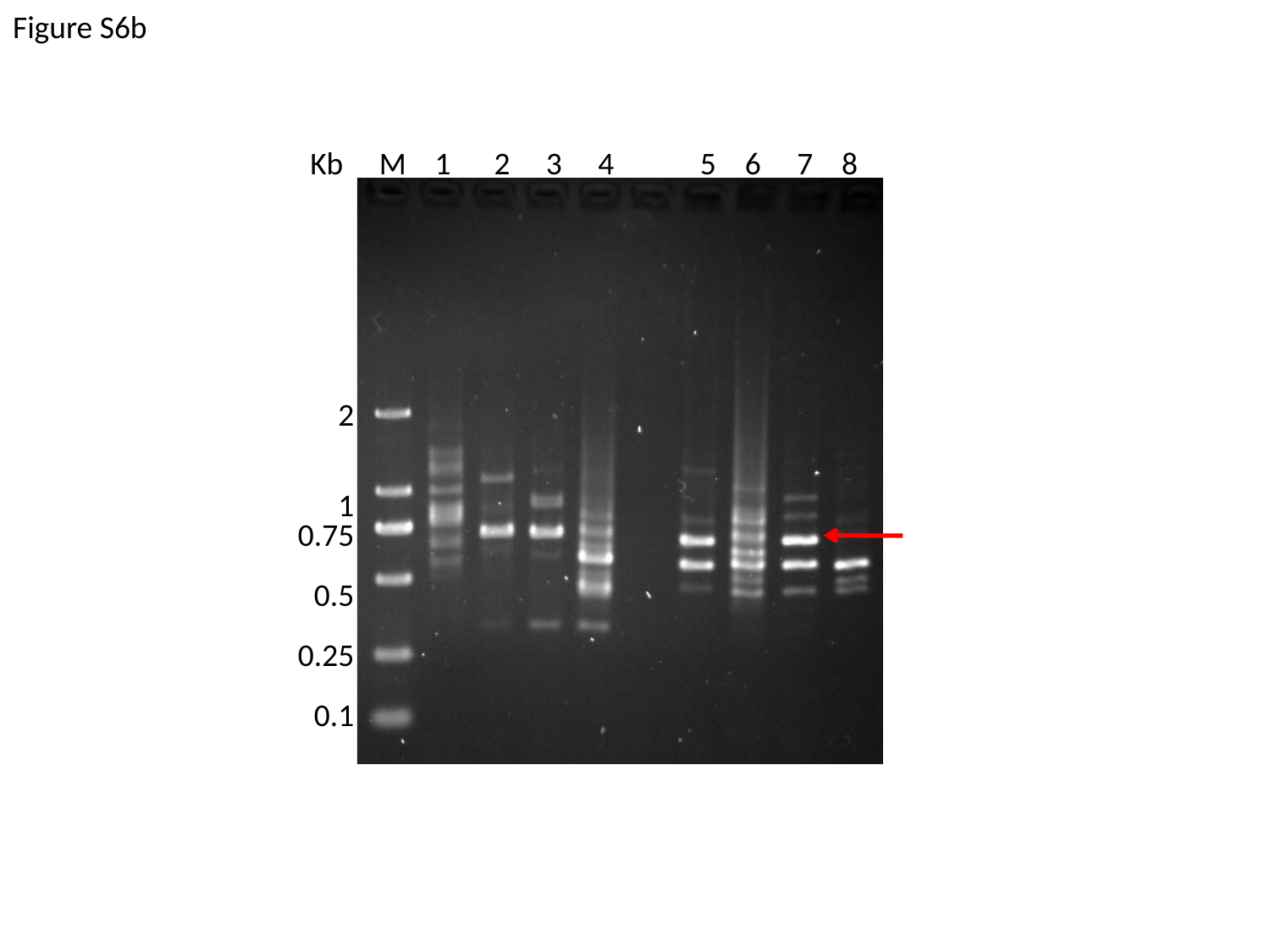

Figure S6b
Kb M 1 2 3 4 5 6 7 8
2
1
0.75
0.5
0.25
0.1

## Slide 8
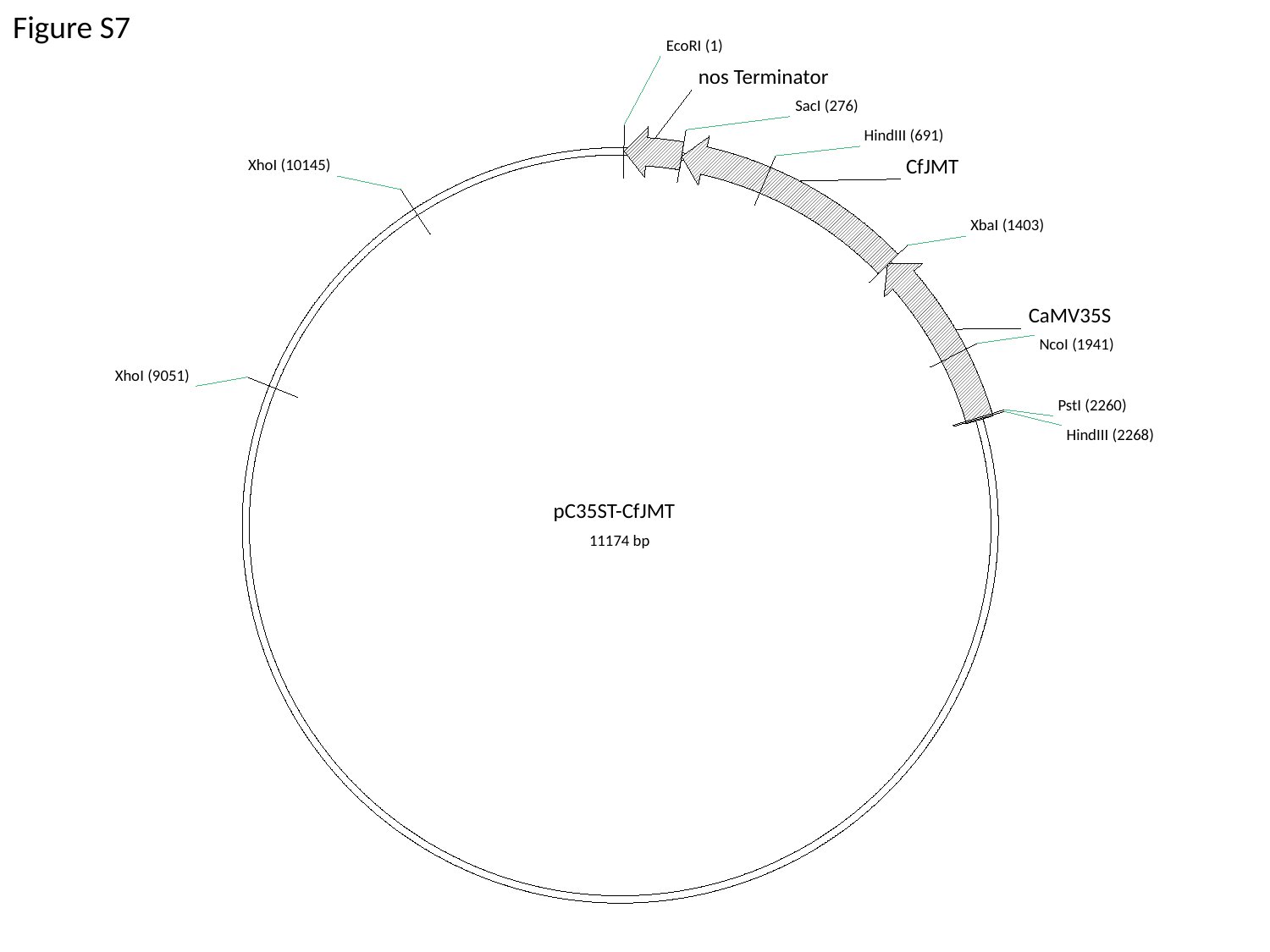

Figure S7
EcoRI (1)
nos Terminator
SacI (276)
HindIII (691)
CfJMT
XhoI (10145)
XbaI (1403)
CaMV35S
NcoI (1941)
XhoI (9051)
PstI (2260)
HindIII (2268)
pC35ST-CfJMT
11174 bp

## Slide 9
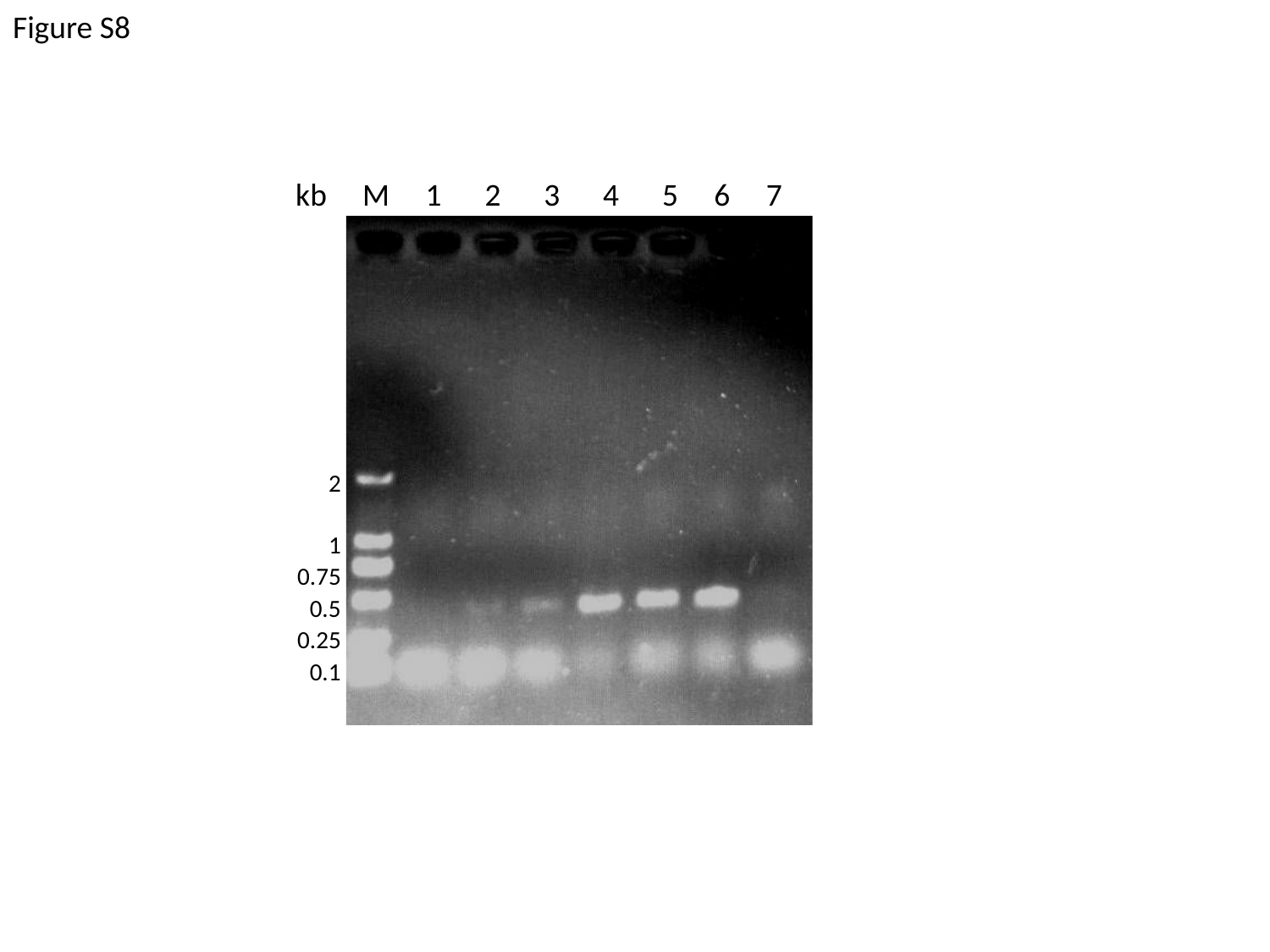

Figure S8
kb M 1 2 3 4 5 6 7
2
1
0.75
0.5
0.25
0.1
