# Supplementary material for: Transcriptomic profiling of the flower scent biosynthesis pathway of Cymbidium faberi Rolfe and functional characterization of its jasmonic acid carboxyl methyltransferase gene
Source: BMC Genomics. 2019 Feb 11;20:125. doi: 10.1186/s12864-019-5501-z (PMC6371524; doi:10.1186/s12864-019-5501-z)
Supplement: Supplementary file 2 — Table S1. Primers derived from unigene sequences used for the verification of the RNA-seq results via qRT-PCR. Table S2. Primers used for TAIL-PCR. (DOCX 15 kb) [file 12864_2019_5501_MOESM2_ESM.docx]

Additional file 2: Table S1 Primers derived from unigene sequences used for the verification of the RNA-seq results via qRT-PCR.

| Name | Sequence (5’→3’) |
| --- | --- |
| Unigene 20155-F | TTCACTTCGACTGCAACTCCTG |
| Unigene 20155-R | TCGTCCTCCCTGTATCCCTTT |
| Unigene 4190-F | CCCATTTGCTGAACATCCTCTG |
| Unigene 4190-R | CAGGCTGGCAGCTTGTATTTG |
| Unigene 12935-F | GCCTTTCCGCCCGTATGC |
| Unigene 12935-R | ATCGCCCGTTACCTGTTGG |
| Unigene 55590-F | TCAGCAAGAAGGATCAAGGG |
| Unigene 55590-R | CCAGCAGATGTGGATTTCG |
| Unigene 43886-F | GGAATCCGACGCCACGAG |
| Unigene 43886-R | CCGCCACGCAACCCATAC |
| Unigene 53798-F | TGCCCAACCTCTAACCACCA |
| Unigene 53798-R | AAGCTGACCGCTGCGACATA |
| Unigene 25970-F | AAGCCATCGCGGTGAAGTT |
| Unigene 25970-R | CCCTTGTTGAGCACTGGAGC |
| Unigene 33715-F | GTCGAGGTCGAGGGGCTTTT |
| Unigene 33715-R | TCGCCGTCTCCACCTCATG |
| Unigene 7762-F | CTCAGCCATAGCAGGAGCCA |
| Unigene 7762-R | GCGACGCTTGCAGAAGGTG |
| Unigene 62274-F | CTTTGCACCTTGCCAACACC |
| Unigene 62274-R | TTCTCCTCCGTCATCCTCCC |

Table S2 Primers used for TAIL-PCR.

| Primer | Sequence (5'→3') |
| --- | --- |
| AD1 | NTCGASTWTSGWGTT |
| AD2 | NGTCGASWGANAWGAA |
| AD3 | WGTGNAGWANCANAGA |
| AD4 | AGWGNAGWANCAWAGG |
| AD5 | NGTAWAASGTNTSCAA |
| AD6 | NGACGASWGANAWGAC |
| AD7 | GTNCGASWCANAWGTT |
| AD8 | NCAGCTWSCTNTSCTT |
| SP1 | GACGCAATAATGCCCTACAG |
| SP2 | GATGATGGAAAAGGTCAGAG |
| SP3 | CGCCGATTCCTCCATTCA |
| 2SP1 | TTAATGGGTTTACTCATTC |
| 2SP2 | CCTCCATTTGACTCTTTACC |
| 2SP3 | TGAATCTTCCCTCCTCGCA |
| 3SP | ACGGAATGTCAGAAATC |

N= G or A or T or C; S= C or G; W= A or T
